# Symmetrical Modification of Minimized Dermaseptins to Extend the Spectrum of Antimicrobials with Endotoxin Neutralization Potency

**DOI:** 10.3390/ijms20061417

**Published:** 2019-03-20

**Authors:** Changxuan Shao, Weizhong Li, Peng Tan, Anshan Shan, Xiujing Dou, Deying Ma, Chunyu Liu

**Affiliations:** 1 Laboratory of Molecular Nutrition and Immunity. The Institute of Animal Nutrition, Northeast Agricultural University, Harbin 150030, China; changxuan_shao@foxmail.com (C.S.); laowu2003@wfu.edu.cn (W.L.); sdwftanpeng@126.com (P.T.); liyang1405053@sina.com (X.D.); liuchunyu@neau.edu.cn (C.L.); 2 College of Biological and Agricultural Engineering, Weifang University, Weifang 261061, China

**Keywords:** antimicrobial peptide, minimized *dermaseptins*, antibacterial mechanism, endotoxin neutralization

## Abstract

Antimicrobial peptides (AMPs) have emerged as a promising class of antimicrobial agents that could potentially address the global antibiotic resistance. Generating mirror-like peptides by minimizing *dermaseptin* family sequences is an effective strategy for designing AMPs. However, the previous research still had some limitations such as lower effectiveness and a narrow spectrum of antibacterial activity. To further expand and hone this strategy, we designed a series of AMPs consisting of the WXMXW-NH_2_ motif (X represents V, I, F, and W; M represents KAAAKAAAK). The peptides formed α-helices and displayed broad-spectrum antimicrobial activities against eleven types of clinical bacteria including both Gram-negative and Gram-positive bacteria. The optimized peptide WW exhibited high physical rupture by inducing membrane shrinkage, disruption, and lysis. Moreover, WW effectively neutralized endotoxins and inhibited the inflammatory response while having the highest therapeutic index. In conclusion, these results indicated that the peptide WW has potential as a broad-spectrum antimicrobial agent or preservative for overcoming the risk of multidrug resistance in localized or external therapeutic applications.

## 1. Introduction

The worldwide challenge for infectious diseases remains exceedingly prominent and has become more disturbing due to the rise of antibiotic resistant bacteria, which is one of the main incentives of social, economic, and public health problems [1]. Antimicrobial peptides (AMPs) play an important role in all multicellular organisms as the first line of the host defense [2]. Unlike conventional antibiotics that cause microbial death by acting on specific cellular targets, the majority of AMPs exert their bioactivities via the non-receptor mediated permeabilization of membranes [3]. The physical disruption of the membrane leads to a low probability of the development of drug resistance as a fundamental change in membrane components is considered to be a slow process [4]. Therefore, AMPs have been found to be equally effective against both antibiotic-sensitive and resistant strains and are expected to become potential substitutes for traditional antibiotics. 

However, the use of AMPs as therapeutic agents has some limitations due to its long peptide sequences, low efficacy, instability, systemic toxicities, and potential for compromising innate host defense immunity, which has hampered the development and clinical application of AMPs [5]. Numerous methods have been developed in the design of analogous peptides to overcome these barriers such as motif hybridization to increase antimicrobial efficacy and functionality [6], truncation/ substitution to decrease toxicity [7], and de novo design to shorten peptide length and eliminate host defense immunogenicity [8]. Nevertheless, these comprehensive strategies represent only a small part of the available methods to promote the development of AMPs. 

In our previous study, “minimized proteins” as an old protein design strategy were newly applied to the design of antimicrobial peptides. According to the *dermaseptin* family sequence and structure information such as charge, amino acid frequency, hydrophobicity, charge ratio, a cluster of Ala in trimer style, and α-helix structure, we designed mirror-like peptides by minimized theory and selected the peptide KL_4_A_6_ (LLKAAAKAAAKLL-NH_2_), which showed the greatest therapeutic index [9]. However, there are still some limitations for KL_4_A_6_ to become a potential therapeutic drug (e.g., narrow spectrum of antibacterial activity and low activity). It has been suggested that tryptophan residues that interact with the membrane at the end of an amphiphilic α-helix will increase antimicrobial activity [10,11], Thus, in this study, short α-helix-folding AMPs (WX) with symmetric sequences, based upon the previous designing principles, consisting of WXMXW (X represents V, I, F, and W; M represents KAAAKAAAK) were designed. The peptide lengths were shortened to 13 residues from 28.75 residues of the *dermaseptin* family, of which three were cationic amino acids (K) and ten were hydrophobic amino acids. The centrosymmetric structure was maintained to increase biological activity [12,13], while different hydrophobic amino acids were used symmetrically for the replacement of residues in the sequence to perturb microbial cell membranes [4,8,14]. Additionally, the C-terminus of the peptide was aminated to enhance antimicrobial activity and improve stabilization [15].

## 2. Results and Discussion

### 2.1. Peptide Design and Characterization

“Minimized proteins” were first proposed in 1997, which represented a potential intermediate step toward the development of drugs targeted to protein–protein interfaces [16]. In fact, the binding surfaces of large protein–protein interactions possess very few residues that confer key functions in the recognition and binding between these proteins. Correspondingly, the interaction between AMP and the bacterial membrane is similar to the interaction between proteins as determinants of the peptide-membrane interaction depend on the physical parameters of AMPs such as charge, amphipathicity, sequence, structure, hydrophobicity, and polar angle [17]. 

The previously designed mirror-like peptide KL_4_A_6_ showed the worst cell selectivity at a therapeutic index (TI) of 9.6, with several limitations. In this study, some rational modification strategies were employed in the optimization of synthetic AMPs to enhance therapeutic potentials including tryptophan residues localizing to the end of an amphiphilic α-helix, centrosymmetric structures, and modifications of different hydrophobic amino acids. The measured molecular weights of each peptide were very close to its theoretical value, suggesting that the products corresponded to the designed compositions (Table 1). Each peptide has four positive charges. 3D hydrophobic moment vectors (3D HMs) are useful as qualitative descriptions of peptide helices, which took into account the cooperation between the side chains and some conformational rearrangements within a whole peptide [18]. The length of 3D HMs for WV, WI, WF, and WW were 36.49, 30.30, 35.79, and 47.47 kTÅ/e, respectively, indicating the following 3D HMs order: WW > WF > WV > WI.

### 2.2. Structure Variability of the Peptides

**Figure 1:**
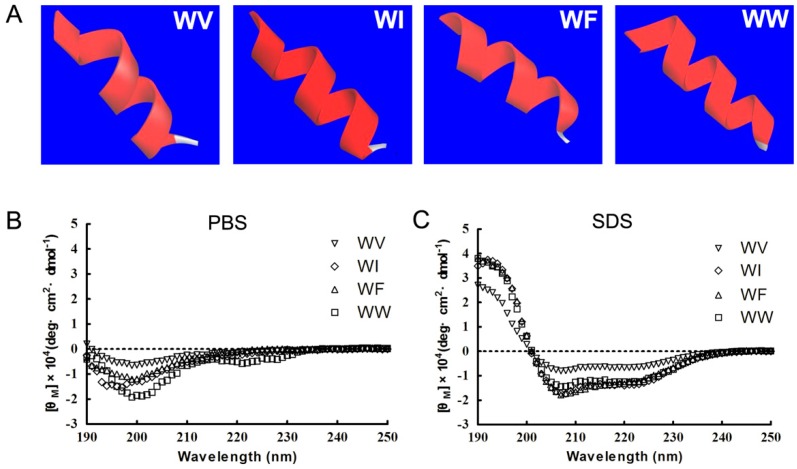
The structure of α-helix forming peptides. (**A**) The 3D structure of the peptides predicted by I-TASSER. The peptides are displayed by a solid ribbon style which was colored online according to secondary structures. red square: α-helix. (**B**,**C**) The CD spectra of the peptides in 10 mM PBS (pH 7.4) (**B**) and 30 mM SDS (**C**). An average of three scans was recorded for each peptide.

As shown in Figure 1A, the designed peptides showed similar α-helices, which was predicted online by computer-aided I-TASSER. These structures were in accordance with the requirement of the *dermaseptin* family structures based on minimized peptide theory. Then, circular dichroism (CD) spectra were measured under 10 mM phosphate-buffered saline (PBS) and 30 mM sodium dodecyl sulfate (SDS). In PBS, the CD spectra of WV, WI, and WF adopted a random coil structure with a minimum at ≈200 nm. The spectrum of WW displayed minima at ≈205 and 220 nm, which supports a disordered structure (Figure 1B). However, in the presence of SDS, the secondary structure of the peptides, with minima at ≈205 and 220 nm, was mainly α-helix (Figure 1C), which indicated that the cationic residues could initiate the association of the peptides onto the surface of the SDS micelles and facilitate the folding of the peptides through electrostatic interactions [19]. Moreover, it has been reported that the conformational transformation can enlarge the area of membrane damage as the peptide approaches the bacterial cell membrane [20].

### 2.3. Antimicrobial Activity

To prove the biological activity of the synthetic peptides, a broad spectrum of model microbes was used in a minimal inhibitory concentration (MIC) assay. As listed in Table 2, the peptides exhibited antibacterial activity against both Gram-negative and Gram-positive pathogens. The geometric means (GM) of the MICs varied from 4.0 to 149.3 μM. Compared with KL_4_A_6_, all peptides, with the exception of WV, showed significantly improved antimicrobial activity and achieved the effective inhibition of *B. pyocyaneum* ATCC 27853, *S. typhimurium* ATCC 14028, *S. pullorum* C79-13, *S. aureus* ATCC 29213, and *S. epidermidis* ATCC 12228. The peptide WW with the lowest GM MIC value of 4.0 μM displayed approximately 1–28 folds higher antimicrobial activity than KL_4_A_6_, indicating the enhanced effectiveness of centrosymmetric Trp modification. It has been previously demonstrated that the Trp residue interacts strongly with the hydrophobic tails of lipids because of its large and bulky indole side chain, which is surrounded by negatively charged π-electron clouds [21], and the Trp residue arranged in the headgroup of the α-helical peptides plays an important role in promoting the biological activity of the peptide [11]. In addition, for the peptide WV, through W residues located at positions 1 and 13 of the sequence, antimicrobial activity was significantly decreased when compared to the peptide KL_4_A_6_. One possible explanation might be that Val has strong β-sheet folding propensity and a negative effect on helical propensity [4]. This phenomenon can be confirmed by CD spectra. The CD spectra of WV showed weak CD signals in the PBS and SDS solution.

### 2.4. Cytotoxicity and Therapeutic Index

**Figure 2:**
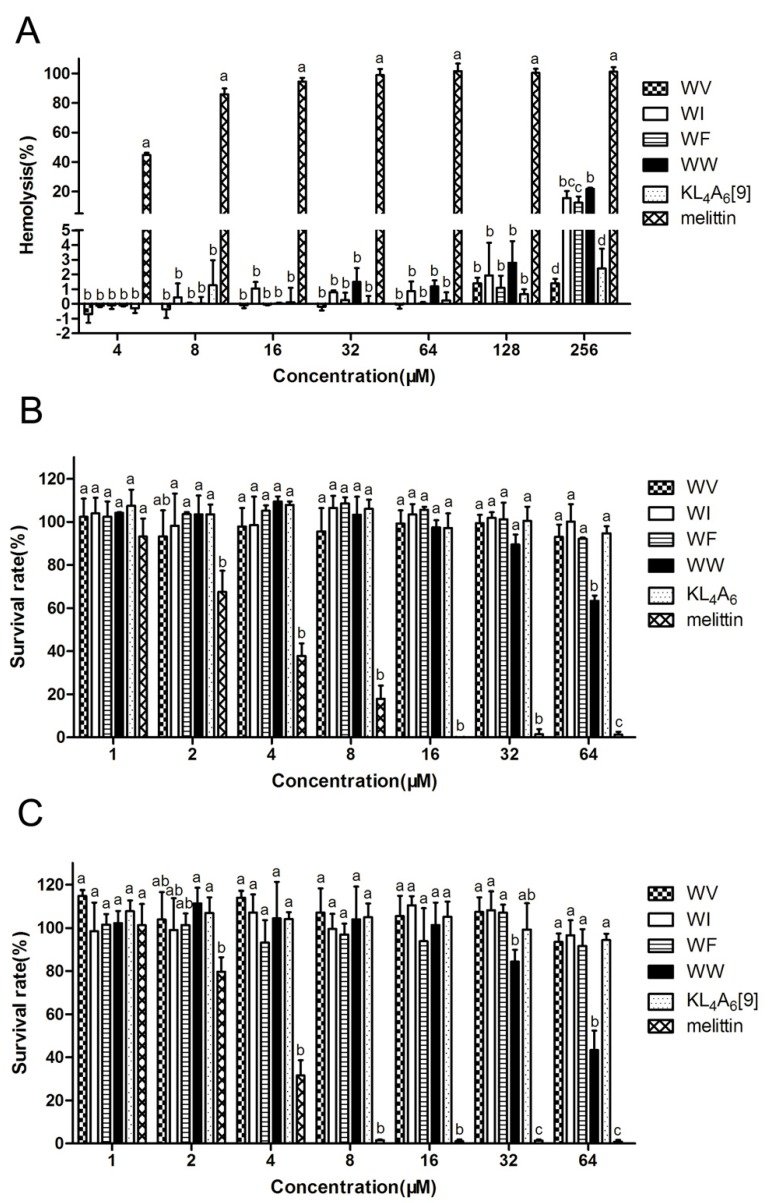
Cytotoxicity of the peptides against human red blood cells (hRBCs) (**A**), RAW 264.7 (**B**), and HEK293T (**C**). Results were given as the mean ± standard deviation (SD) of three independent trials. Differences between groups exposed to the same concentration were determined by one-way ANOVA followed by Turkey’s post-hoc analysis (*n* = 3). The values with different superscripts (a, b, c, d) indicate a significant difference (*p* < 0.05).

According to Table 2 and Figure 2A, dose-response studies revealed that hemolytic activity was relative to different hydrophobic residues in WX peptides at the higher concentration. On the whole, the results showed that four WX peptides had remarkably lower cytotoxicity and hemolysis when compared with melittin (the control). At the highest concentration (256 μM), the hemolysis of the peptides was as follows: WW > WI > WF > WV, and was higher than that of the peptide KL_4_A_6_ [9], suggesting an important role of the tryptophan residues residing at the end of an amphophilic alpha-helix for hemolytic activity. The results showed that the cell survival rate of the murine macrophage cell line (RAW264.7) at a peptide concentration of 64 μM was as follows: WW (63%) < WF (92%) < WV (93%) < WI (100%) (Figure 2B). Similarly, the cell survival rate of human embryonic kidney cells (HEK293T) at a peptide concentration of 64 μM was WW (43%) < WF (91%) < WV (93%) < WI (96%) (Figure 2C). It should also be noted that WW was significantly more toxic to both cells than KL_4_A_6_ and the other three WX-series peptides at 64 μM. Thus, the modification of aromatic residues such as Phe and Trp had higher hemolytic activity and cytotoxicity than that of Ile or Val. This was related to the fact that aromatic residues are considered to have a pivotal function on the partition by anchoring the peptide to the membrane [11]. However, taking antimicrobial efficacy into consideration, WW showed the greatest selectivity toward bacteria over mammalian cells with a TI value of 64.0. Therefore, WW was selected to explore the mechanism of action.

### 2.5. Mechanism of Action of the Peptides

**Figure 3:**
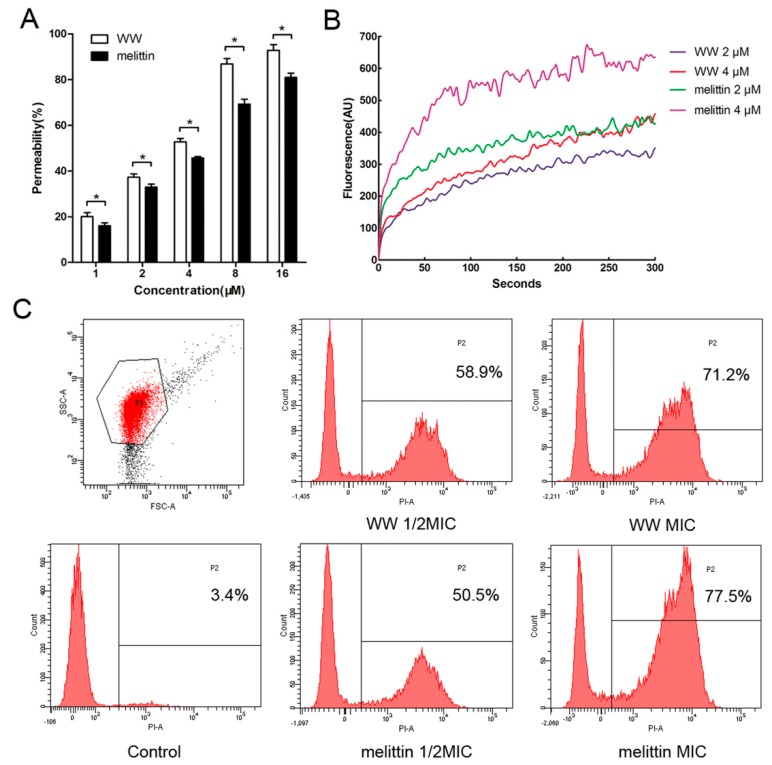
The influence of WW and melittin on the outer membrane permeability (**A**), cytoplasmic membrane depolarization (**B**), and membrane integrity (**C**). Results in (**A**) are given as the mean ± standard deviation (SD) of three independent experiments and the difference (* *p* < 0.05) between two groups exposed to the same peptide concentration was analyzed by the Student’s *t* test. The results in (**B**) were expressed as an average of three independent trials. The data in (**C**) are representative of three independent experiments.

Compared to conventional antibiotics, most AMPs kill bacteria through the disruption of the microbial membrane [22,23]. Gram-positive bacteria and Gram-negative bacteria share the commonality of having a cytoplasmic membrane. Gram-negative bacteria are surrounded by an extra outer membrane (OM) containing lipopolysaccharide (LPS) as the major lipid component, which performs the crucial role of compromising the exchange of material required for sustaining life [24]. Figure 3A shows that the dose-response relationship between OM permeability and concentration (1–16 μM) of the peptides. At a concentration of 16 μM, the two peptides caused an outer membrane permeability greater than 75%. Interestingly, the outer membrane permeability of WW was stronger than those of melittin at the same concentration, even though WW had a lower antibacterial activity against *E. coli* UB1005 than melittin.

Thus, upon the permeabilization of the outer membrane, the electrical potential changes of the cytoplasmic membrane were further measured by 3,3′-dipropylthiadicarbo-cyanine iodide (diSC_3_-5). The bacterial cytoplasmic membrane consists predominantly of hydroxylated phospholipids (e.g., cardiolipin, phosphatidylglycerol, phosphatidylserine), representing a negative net charge at physiological pH [25], which enhance the binding of the cationic AMPs. Upon membrane absorption and binding, the peptide was then inserted into the lipid bilayer, resulting in membrane permeabilization and pore/ion channel formation, simultaneously concomitant with the membrane potential collapse. As shown in Figure 3B, these two peptides induced a sustained release of diSC_3_-5 over a period of 300 s, and every peptide produced dose-dependent results at the final time of 300 s. Melittin induced a higher and faster increase of fluorescence than those of WW at 2 μM and 4 μM, respectively. Considering the moderate OM permeabilization of melittin, we reasoned that the stronger antimicrobial activity against *E. coli* UB1005 by melittin than WW may be due to its higher cytoplasmic membrane depolarization. 

Furthermore, fluorescence-assisted cell sorting (FACScan) analysis was performed to determine the membrane integrity of *E. coli* treated with WW or melittin as the fluorescent probe propidium iodide (PI) can incorporate and stain nucleic acids in cells with damaged membranes [26]. The data were then analyzed once and displayed as two-parameters, complexity (Side Scatter, SSC) and cell size (Forward Scatter, FSC) before the fluorescence frequency distribution histogram of the *E. coli* population was obtained (P1). As shown in Figure 3C, the *x*-axis represents the fluorescent emission of PI, and the count (*y*-axis) of PI-positive cells was measured for each peptide concentration. The data showed that the percentage of PI-positive cells with membranes damaged by WW was 58.9% (1/2 × MIC) and 71.2% (1 × MIC). The percentage of PI-positive cells treated with melittin was 50.5% (1/2 × MIC) and 77.5% (1 × MIC). This observation indicated that WW has the ability to permeabilize the cytoplamic membranes of bacteria, similar to melittin, which results in cell death. 

### 2.6. Membrane Morphological Analysis

**Figure 4:**
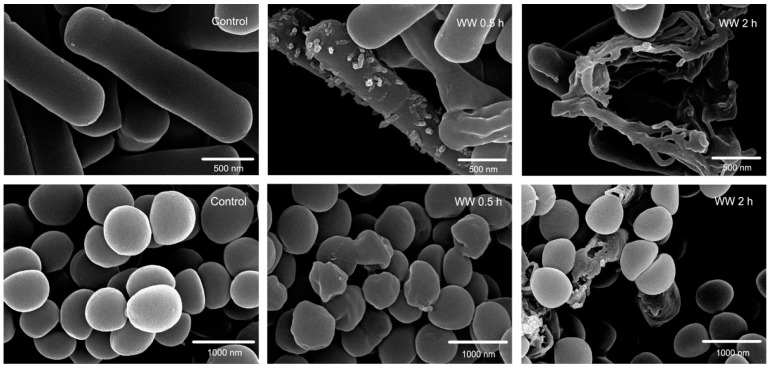
Scanning electron micrographs of *E. coli* 25922 and *S. aureus* 29213 treated with WW at 1 × MIC. All data are representative of three independent experiments.

**Figure 5:**
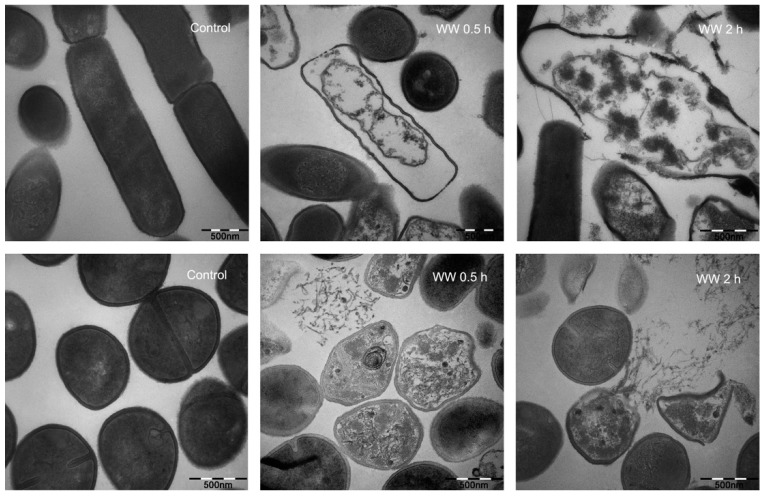
Transmission electron micrographs of *E. coli* 25922 and *S. aureus* 29213 treated with WW at 1 × MIC. All data are representative of three independent experiments.

Based on the rapid permeabilization and depolarization of microbial membranes, we visualized the bacterial membrane damage by field emission scanning electron microscopy (FE-SEM) and transmission electron microscopy (TEM). Figure 4 and Figure 5 demonstrate that membrane morphology of the cells treated with the peptides were all obviously different from the control (no peptide). Under FE-SEM, the controls showed a smooth and intact surface. After treatment with the peptide WW for 30 min, the membrane surface morphology and integrity of both the Gram-negative *E. coli* and Gram-positive *S. aureus* were observed as a rough, shrunken, and slight leakage of cellular cytoplasmic content to cell lysis, resulting in the formation of visible membrane atrophy and corrugation. After 2 h of WW treatment, significant membrane rupture occurred in the bacteria, some of which even appeared to be completely dissolved. Similarly, after 30 min, peptide WW induced significant morphological and intracellular changes of *E. coli* and *S. aureus* when compared to a control with a smooth cell membrane and homogeneous cytoplasm under TEM, and caused severe membrane lysis and plasmolysis after 2 h. This observation is consistent with the documented membrane lysis mechanism of KL_4_A_6_ [9] and some other natural or synthetic AMPs [27]. 

### 2.7. Endotoxin Neutralization Assay

**Figure 6:**
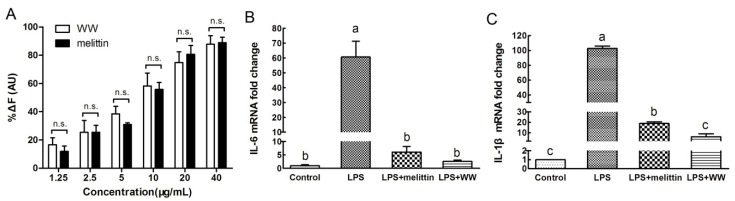
(**A**) Peptide binding affinity to LPS from *E. coli* 0111:B4. Results in Figure 6A are given as mean ± standard deviation (SD) of three independent experiments and the difference (n.s., not significant at *p* < 0.05) between two groups exposed to the same peptide concentration was analyzed by the Student’s *t* test. (**B**,**C**) WW inhibited the LPS-induced gene expression of pro-inflammatory cytokine effects in the murine macrophage cell line RAW264.7. Results are given as the mean ± SD. Differences between groups were determined by one-way ANOVA followed by Turkey’s post-hoc analysis (*n* = 3). The values with different superscripts (a, b, c) indicated a significant difference (*p* < 0.05).

As we know, LPS, as a major component of the outer membrane of Gram-negative bacteria, can increase the expression of pro-inflammatory cytokines by binding to its receptors, thereby triggering a systemic inflammatory response [28]. With the continuous advancement of the properties and performance of antimicrobial peptides in the past two decades, in addition to classical antibacterial activity, they can also be used as antitoxin drugs to bind and isolate LPS [29]. In this study, we first assessed the binding activity of WW to LPS (100 ng/mL) by using a fluorescence-based displacement assay with BODIPY-TR cadaverine (BC). As shown in Figure 6A, both WW and melittin displayed a concentration-dependent increase in the fluorescent intensities. At all concentrations, the %ΔF (AU) caused by WW and melittin did not show significant differences. However, at low concentrations (1.25, 2.5, 5, 10 μg/mL), the changes in fluorescence intensity caused by WW were slightly higher than that of melittin. As the peptide concentration increased, the binding of melittin to LPS showed a stronger increase than WW. 

Subsequently, the gene expression of pro-inflammatory cytokines (e.g., interleukin-6(IL-6) and interleukin-1β(IL-1β)) in macrophages was measured to determine the inhibitory effect of WW on the LPS-induced inflammatory response. As shown in Figure 6B,C, untreated cells and LPS-alone (100 ng·mL^−1^) treated cells served as the negative and positive controls, respectively. After stimulation with LPS, the gene expression of IL-6 and IL-1β from the murine macrophages were robustly increased. However, in the presence of WW or melittin, the gene expression of pro-inflammatory factors in cells treated with LPS were markedly decreased. WW was more effective than melittin to attenuate the mRNA expression of IL-1β. Thus, the peptide WW has the potential to maintain the balance of cytokines in the inflammatory response induced by LPS. 

## 3. Materials and Methods 

### 3.1. Bacterial Strains and Mammalian Cells

The bacterial strains *E. coli* ATCC25922, *P. aeruginosa* ATCC27853, *S. typhimurium* ATCC14028, *S. pullorum* C7913, *S. aureus* ATCC29213, *S. epidermidis* ATCC12228, *S. faecalis* ATCC29212, and the model microbe *B. subtilis* CMCC63501 were obtained from the College of Veterinary Medicine, Northeast Agricultural University (Harbin, China). *E. coli* UB1005 was provided by the State Key Laboratory of Microbial Technology, Shandong University (Jinan, China). The hRBCs were obtained from the Northeast Agricultural University Hospital. RAW264.7 was purchased from the cell bank of the Chinese Academy of Sciences, SIBS (Shanghai, China). HEK293T was obtained from the College of Animal Science and Technology, Northeast Agricultural University (Harbin, China). 

### 3.2. Peptides Synthesis and Sequence Analysis

All peptide-based solid phase protocols were synthesized by GL Biochem (Shanghai, China), and identified via matrix-assisted laser desorption/ionization time-of-flight mass spectrometry (MALDI-TOF MS, Linear Scientific Inc., Billerica, MA, USA.) using α-cyano-4-hydroxycinnamic acid (HCCA) as the matrix. Analytical reversed-phase high-performance liquid chromatography (RP-HPLC) (LC 3000, Beijing, China) was used to confirm the purity of each peptide (>95%). The powder form peptide was dissolved in deionized water at a concentration of 2560 μM and stored at −20 °C before subsequent evaluations. The primary physicochemical parameters were calculated using the AMP database [30]. The 3D structure projection was predicted online by I-TASSER [31]. 3D hydrophobic moment (HM) vectors were calculated online by a 3D-HM calculator [32].

### 3.3. CD Spectroscopy

The CD spectra were performed on a J-820 spectrometer (Jasco, Tokyo, Japan). Data were recorded at 25 °C with the following parameters: 0.1 cm path length, 10 nm∙min^−1^ scanning rate, and 190 to 250 nm wavelength range. The final concentration of the peptide samples was 150 mM in 10 mM PBS to mimic the aqueous environment and in 30 mM SDS micelles to mimic the negatively charged prokaryotic membrane comparable environment. The acquired data were converted to mean residue ellipticity as follows: θ_M_ = (θ_obs_ × 1000)/(l × c × n), where θ_M_ is the mean residue ellipticity (deg∙cm^2^∙dmol^−1^), θ_obs_ is the measured ellipticity corrected for the buffer at a given wavelength (mdeg), l is the path length (cm), c is the peptide concentration (mM), and n is the number of amino residues.

### 3.4. MIC Measurements

MIC measurements were performed in 96-well plates [33]. The peptides were dissolved in 0.2% bovine serum albumin and 0.01% acetic acid, and an appropriate broth was subjected to a series of two-fold dilutions. The final concentration of microbial suspensions was from 2 × 10^5^ to 7 × 10^5^ colony forming units (CFU)∙mL^−1^. Key parameters were volume (50 μL bacterial suspension + 50 μL peptide solution), incubating time (18–24 h), and temperature (37 °C). Each test was performed in three replicates on at least two independent occasions. The Mueller Hilton broth (MHB) (AoBoX, Shanghai, China) without or with microbial cells was regarded as the negative control or positive control, respectively. MICs were recorded at the lowest peptide concentration without visible turbidity and spectrophotometrically by measuring the optical density (OD) at 492 nm (Tecan, Salzburg, Austria). 

### 3.5. Hemolytic Activity Assay

Hemolytic activity measurements were performed to evaluate the effect of peptides on hemoglobin release levels by erythrocyte lysis [34]. The experimental protocol was reviewed and approved by the ethics committee of the Northeast Agricultural University Hospital, and the experimental method was carried out in accordance with the approved guidelines and regulations. A total of 1 mL of fresh hRBCs was collected and diluted 10-fold with PBS (pH 7.4). A quantity of 50 μL of the hRBCs solution was incubated with 50 μL of serially diluted peptides dissolved in PBS for 1 h at 37 °C. hRBC suspension treated with 0.1% Triton X-100 was employed as a positive control and an untreated hRBC suspension was used as a negative control. A total of 50 μL of the supernatant was collected from the mixture after centrifugation (1000*g*, 5 min, 4 °C) and transferred to a new 96-well microplate. The percent hemolysis was calculated using the following equation: Hemolysis (%) = [(OD570 of the treated sample — OD570 of the negative control)/(OD570 of the positive control — OD570 of the negative control)] × 100%.

### 3.6. Cytotoxicity Assay

The Roswell Park Memorial Institute-1640 (RPMI-1640) (Invitrogen, Carlsbad, CA, USA) and Dulbecco’s modified Eagle’s medium-high glucose (DMEM) (Gibco, China) were supplemented with 100 μg∙mL^−1^ streptomycin sulfate, 100 U∙mL^−1^ penicillin, and 10% (v/v) fetal bovine serum (Hyclone, Logan, UT, USA). These were used to culture the RAW264.7 and HEK293T cells, respectively. The peptides were added to cell cultures (1.0–2.0 × 10^4^ cells/well) at different final concentrations (1–64 µM) in 96-well plates for 18–24 h at 37 °C in 5% CO_2_, and 3-(4,5-dimethylthiazol-2-yl)-2,5-diphenyltetrazolium bromide (MTT) (50 µL, 0.5 mg∙mL^−1^) was injected and incubated with cell cultures for 4 h at 37 °C. Finally, the centrifuged cells without the supernatants were mixed with 150 µL of dimethyl sulfoxide to dissolve the formazan crystals that formed. The optical density (OD) was recorded by a microplate reader (Tecan, Salzburg, Austria) at 570 nm.

### 3.7. Outer Membrane (OM) Permeability Assay

An OM permeability assay was performed on an F-4500 fluorescence spectrophotometer (Hitachi, Japan). Prior to the experiment, *E. coli* UB1005 in mid-log phase was obtained to wash thrice with 4-(2-hydroxyethyl)piperazine-1-ethanesulfonic acid (HEPES) buffer (pH 7.4, containing 5 mM glucose) and diluted to 10^5^ CFU∙mL^−1^ in the same buffer. Data were recorded (excitation λ = 350 nm, emission λ = 420 nm) in a quartz cuvette with a 2 mL bacterial suspension containing 10 μM N-phenyl-1-naphthylamine (NPN). The observed values were converted to % NPN uptake using the equation:

% NPN uptake = (*F_obs_* − *F_0_*) / (*F_100_* − *F_0_*) × 100

where *F_0_* is the background fluorescence of NPN in the absence of peptide; *F_100_* is the fluorescence of NPN upon addition of 10 μg∙mL^−1^ polymyxin B (positive control); and *F_obs_* is the measured fluorescence at a given peptide concentration.

### 3.8. Cytoplasmic Membrane Depolarization Assay

A membrane potential-sensitive fluorescent dye, diSC_3_-5, was used to determine the cytoplasmic membrane depolarization activity of the peptides. Coalescent preparation of *E. coli* UB1005 suspension was performed in different HEPES buffer (pH 7.2, 5 mM HEPES buffer, 20 mM glucose, 100 mM KCl) and reached an OD_600_ of 0.05. Data were recorded (excitation λ = 622 nm, emission λ = 670 nm) in a quartz cuvette with a 2 mL bacterial suspension containing different concentrations of peptides after incubation with 0.4 μM diSC_3_-5 for 1 h at 37 °C. Observation changes were recorded from 0 to 300 s when the cell suspension was mixed with peptides at final concentrations of 2 μM and 4 μM, respectively. 

### 3.9. FACScan Analysis

The membrane integrity measurements were performed with a FACScan instrument (Bectone-Dickinson, San Jose, CA, USA) by using a modified method as described previously [6]. *E. coli* ATCC 25922 was similarly prepared to the MIC measurements, washed thrice with PBS, and diluted to 10^5^ CFU·mL^−1^ in the same buffer. Two concentrations of the peptides (1/2 and 1 × MIC) were added to the bacterial suspension containing 10 μg·mL^−1^ propidium iodide (PI) at 4 °C for 30 min. As a control, bacteria were exposed to PBS without the peptide. The gate (P1) was defined based on the FSC and SSC properties of *E. coli.* The FSC threshold was appropriately raised to include the large cell events in the sample and excluded the debris population. Data acquisition was performed using FACSDIVA Software (Becton Dickinson, San Jose, CA, USA) with a stopping gate of 10,000 total events. Observations were repeated three times at a laser excitation wavelength of 488 nm. Based on the ability of flow cytometers to provide accurate quantification of cell numbers, the counts of PI-positive cells were measured for each peptide. 

### 3.10. Membrane Morphological Observations

The preparation of the bacteria suspension was similar to the FACScan analysis. The cells were incubated with the peptide (0 and 1 × MIC) at 37 °C for 30 and 120 min, respectively. After incubation, the cells were centrifuged (5000× *g*, 5 min) and washed three times with PBS (10 mM). Harvested cell pellets were subjected to fixation with glutaraldehyde (2.5%) for 6 to 8 h at 4 °C, followed by washing twice with PBS. Furthermore, the cells were dehydrated for 10 min in a graded ethanol series (50%, 70%, 90%, and 100%), followed by 15 min in 100% ethanol. Then, the specimens for SEM were dehydrated for 15 min in a mixture (v:v = 1:1) of 100% ethanol and tertiary butanol, and absolute tertiary butanol. Finally, the specimens were dried in a critical point dryer with liquid CO_2_, coated with gold-palladium, and then observed using a Hitachi S-4800 SEM (Hitachi, Japan). The TEM samples were dehydrated in a mixture (v:v = 1:1) of 100% ethanol and acetone and absolute acetone for 15 min, and then transferred to a 1:1 mixture of absolute acetone and epoxy resin for 30 min and then to pure epoxy resin and were incubated overnight at a constant temperature. Finally, the specimens were sectioned with an ultramicrotome, stained with uranyl acetate and lead citrate, and observed using a Hitachi H-7650 TEM (Hitachi, Japan). 

### 3.11. LPS Neutralization Assay

BODIPY-TR-cadaverine fluorescent dye (BC, Sigma, USA) was used to determine the LPS neutralizing activity of the peptides. A total of 100 ng/mL LPS from *E. coli* O111:B4 was incubated with 5 μg/mL BC in Tris buffer (50 mM, pH 7.4) for 4 h at room temperature. The peptides were serially diluted to different concentrations (1.25–40 μg/mL) in a 96-well plate and incubated with an equal volume of the LPS-probe mixture for 1 h at 37 °C. The fluorescence was recorded (excitation λ = 580 nm, emission λ = 620 nm) with a spectrofluorophotometer (the Infinite 200 pro, Tecan, China). The values were converted to %ΔF (AU) using the following equation:

%ΔF (AU) = (*F_obs_* − *F_0_*) / (*F_100_* − *F_0_*) × 100

where *F_0_* is the initial fluorescence of BC with LPS; *F_100_* is the BC fluorescence with LPS cells upon the addition of 10 μg∙mL^−1^ polymyxin B (the positive control); and *F_obs_* is the observed fluorescence at a given peptide concentration. 

### 3.12. Cytokine Measurements

The RAW264.7 cell culture was prepared using the same method as the cytotoxicity assay. The cells were seeded in 12 wells plates at a given density (5 × 10^5^ cells per well); after overnight culture, 100 ng·mL^−1^ LPS from *E. coli* 0111:B4 was added to stimulate the generation of cytokines in the presence or absence of the peptides at concentrations of 10 μg·mL^−1^. The mRNA levels of IL-6 and IL-1β were determined by quantitative real-time PCR(qRT-PCR) as previously described [33]. The total RNA was isolated from the RAW264.7 cells using TRIzol according to the manufacturer’s instructions. Total RNA (1 µg) was reverse transcribed by the M-MLV Reverse Transcriptase (Thermo scientific, Waltham, MA, USA). The reactions were incubated in chronological arrangement with the following parameters: at 25 °C for 20 min, at 42 °C for 60 min, and at 70 °C for 10 min. The obtained cDNA samples were determined by qRT-PCR with SYBR green I fluorescence dye (Takara Bio Inc., Kusatsu, Japan). Relative quantifications of the mRNA expression of the target genes were calculated through the comparative threshold cycle number for each sample (2^−ΔΔCT^). Gene expression was normalized to the corresponding β-actin level. The primers for qRT-PCR in this study are shown below: IL-6-F: ggagaggagacttcacagagga; IL-6-R: atttccacgatttcccagaga; IL-1β-F: tgaaatgccaccttttgacag; and IL-1β-R: ccacagccacaatgagtgatac.

### 3.13. Statistical Analysis

Data were analyzed by the unpaired Student’s *t*-test or one-way ANOVA using SPSS 22.0 software (IBM, Chicago, IL, USA). Quantitative data were presented as the mean ± standard deviation of the mean. Differences were defined as significant when the P-value was less than 0.05. 

## 4. Conclusions

In this study, the minimized *dermaseptin* peptide was optimized by symmetry modification in light of the physicochemical properties. The derivatives were folded into α-helical structures in membrane-mimetic environments, but were unstructured in water. Optimized peptides demonstrated an enhanced and extended spectrum of antimicrobial activities against both Gram-positive and Gram-negative bacteria. The peptide WW was found to possess a high cell selectivity with TI = 64.0. WW exhibited the ability to permeabilize the outer membrane of bacterial cells and to depolarize the cytoplasmic membrane, which damaged the membrane integrity and caused intracellular content leakage. Additionally, it displayed the capacity to modulate LPS-induced inflammatory response in RAW264.7 cells by avoiding the overexpression of cytokines. In short, all of the data we presented are helpful in the design of AMPs and the centrosymmetric modification of minimized peptide WW has potential as a broad-spectrum antimicrobial, antisepsis, or topical endotoxin neutralization agent.

## Figures and Tables

**Table 1:** Peptides design and key physicochemical parameters.

Peptides	Sequences	Molecular Mass		Net Charge	3D Hydrophobic Moment Vectors (20.0) ^1^	
		Calculated	Measured		Length (kTÅ/e)	Angle (°)
KL_4_A_6_ [9]	LLKAAAKAAAKLL-NH_2_	1280.69	1280.68	+4	18.94	109.81
WV	WVKAAAKAAAKVW-NH_2_	1398.74	1398.73	+4	36.49	30.87
WI	WIKAAAKAAAKIW-NH_2_	1426.79	1426.78	+4	30.30	141.05
WF	WFKAAAKAAAKFW-NH_2_	1494.83	1494.82	+4	35.79	86.60
WW	WWKAAAKAAAKWW-NH_2_	1572.87	1572.86	+4	47.40	84.50

^1^ 20.0 is the solvent dielectric constants used for the membrane-water interface.

**Table 2:** MICs and therapeutic index of the peptides.

MIC (μM)	KL_4_A_6_ [9]	WV	WI	WF	WW	Melittin
Gram-negative bacteria
*E. coli* ATCC25922	2	32	8	4	2	1
*E. coli* UB1005	32	>128	8	4	4	2
*P. aeruginosa* ATCC 27853	>128	>128	32	16	8	2
*S. typhimurium* ATCC 14028	128	>128	32	32	8	4
*S. pullorum* C79-13	>128	64	8	8	2	1
Gram-positive bacteria
*S. aureus* ATCC 29213	>128	>128	32	8	4	1
*S. epidermidis* ATCC 12228	128	>128	32	16	4	0.5
*S. faecalis* ATCC 29212	16	64	32	32	4	1
*B. subtilis* CMCC 63501	32	>128	8	8	4	1
MHC_5_(μM) ^1^	>256	>256	256	256	256	0.25
GM(μM) ^2^	59.3	149.3	17.3	10.9	4.0	1.3
Therapeutic index ^3^	8.6	3.4	14.8	23.5	64.0	0.2

^1^ Minimum hemolysis concentration 5% (MHC_5_) is defined as the lowest peptide concentration that induces 5% hemolysis. When no detectable hemolytic activity was observed at 256 μM, a value of 512 μM was used to calculate the therapeutic index. ^2^ Geometric mean (GM) of MIC values for the 10 bacteria were tested. When no detectable antimicrobial activity was observed at 128 μM, a value of 256 μM was used to calculate the therapeutic index. ^3^ Therapeutic index (TI) was calculated as MHC_5_/GM.
